# Understanding Patient Portal Uses and Needs: Cross-Sectional Study in a State Fair Setting

**DOI:** 10.2196/64085

**Published:** 2024-10-11

**Authors:** Sripriya Rajamani, Robin Austin, Chelsea Richwine, Malin Britt-Lalich, Madhur Thakur, Yasmin Odowa, Ratchada Jantraporn, Jenna Marquard

**Affiliations:** 1 University of Minnesota Minneapolis, MN United States; 2 Office of the Assistant Secretary for Technology Policy, US Department of Health and Human Services Washington, DC United States

**Keywords:** patient portals, patient engagement, health information technology, consumer health informatics, health informatics, use, online access, medical records, data access, functionality

## Abstract

This study identified 22 features that are used and the needs for desired features/data in patient portals that enable online access to medical records. Data collected at a Midwestern state fair indicates that while most participants used patient portals, use and desirability of specific features varied widely. Identified needs for enhanced data access, portal functionality, and usability can be used to inform effective patient portal design.

## Introduction

There is increasing interest in patient engagement via consumer-facing digital health tools such as patient portals that enable online access to medical records [1-3]. Federal policies such as the Electronic Health Record Incentive Program [4] and the Office of the National Coordinator for Health Information Technology’s Cures Act Final Rule [5] have been instrumental in advancing patient access to electronic health information (EHI). Despite growth in patient portal access [6], there is variation in the use of features [7] and a limited understanding of desired features and data needs. This study contributes to the literature by examining the use and interest in available portal features and identifying desired features and unmet data needs.

## Methods

### Overview

Data collection occurred at the Minnesota State Fair as part of the University of Minnesota’s Driven-to-Discover Initiative in August-September 2023 [8] (Multimedia Appendix 1). Participants completed a Qualtrics survey in English on iPads. The survey was developed by the authors and included select questions from the Health Information National Trends Survey. Participants were first asked about their frequency of logging into their patient portal in the past 12 months and, among those indicating no use, reasons for not accessing their portal. Respondents were then asked about their use and interest in 22 patient portal features identified in the literature and those available in Epic MyChart, the leading electronic health record vendor in the state. Features were organized into the following categories: viewing information, finding care, communication, billing and insurance, and sharing and updating information. For each feature, respondents were asked to indicate whether this was a feature they have used, have not used but are interested in using, have not used and are not interested in using, or did not have available. The survey also included questions on desired portal features and data needs related to patient portals (Multimedia Appendix 2). In this study, participants’ frequency of accessing their portal, reasons for nonuse, and use and interest in specific portal features are described, and desired features and data needs related to patient portals are identified. All data were analyzed using SPSS (v28; IBM Corp; Multimedia Appendix 3).

### Ethical Considerations

This research was approved by the University of Minnesota’s Institutional Review Board (STUDY00019153). An informed consent was solicited at the start of the survey (Multimedia Appendix 2), and participants received a backpack (US $2 value) as an incentive. Study data are anonymous, and no individual identifying information was collected.

## Results

The participants (N=523) included were adults (aged >18 years), and most were current portal users (n=465, 88.9%). Portal users were White (n=375, 80.6%), non-Hispanic (n=414, 89%), female (n=325, 69.9%), and married (n=233, 50.1%). Most participants had a college or postgraduate education (n=339, 72.9%), resided in a suburban/urban area (n=408, 87.7%), and lived comfortably on their present income (n=313, 67.3%).

Of the 465 portal users, close to two-thirds (n=283, 60.9%) accessed their patient portal more than five times in the past 12 months, indicating frequent use. Notably, the portal nonusers (n=58, 11.1% of the 523 participants) had zero logins, and the reasons noted were lack of portal access, no perceived need, discomfort with computers, privacy/security concerns, and combined reasons, highlighting diverse factors influencing utilization.

For patients who accessed their portal at least once in the past 12 months, Table 1 shows that the frequently used features were viewing lab results (n=431, 92.7%) and visit information (n=412, 88.6%). The frequent features of interest but that are not currently used were pulling information from the state immunization registry into their portal (n=283, 60.9%) and entering vaccination information (n=274, 58.9%).

Table 2 displays the responses for desired portal features, with the most common being the ability to share genetic testing information with providers (n=343, 73.8%) and access to test results before providers review them (n=341, 73.3%). The most common data needs were data to be explained more clearly (n=157, 33.8%) and the need for more data from providers (n=135, 29%).

**Table 1:** Use of features and interest in using features in patient portals among 465 portal users.

Features		Have used, n (%)	Have NOT used, but interested in using, n (%)	Have NOT used, and NOT interested in using, n (%)	Feature not available, n (%)
**Viewing information**					
	View laboratory results	431 (92.7)	25 (5.4)	9 (1.9)	0 (0.0)
	View prior and upcoming visit information	412 (88.6)	41 (8.8)	10 (2.2)	2 (0.4)
	Complete questionnaires and forms	351 (75.5)	73 (15.7)	34 (7.3)	7 (1.5)
	View vaccinations	337 (72.5)	103 (22.2)	18 (3.9)	7 (1.5)
	View medications	324 (69.7)	110 (23.7)	26 (5.6)	5 (1.1)
	Complete advanced care planning	159 (34.2)	212 (45.6)	69 (14.8)	25 (5.4)
**Finding care**					
	Schedule nonurgent appointment	345 (74.2)	75 (16.1)	34 (7.3)	11 (2.4)
	Schedule e-visit, telehealth, or video visit	247 (53.1)	125 (26.9)	79 (17.0)	34 (3.0)
	Schedule urgent visit for a health condition	181 (38.9)	186 (40.0)	79 (17.0)	19 (4.1)
**Communication**					
	Ask question to doctor/nurse/care team	333 (71.6)	107 (23.0)	21 (4.5)	4 (0.9)
	Provide information to doctor/nurse/care team	298 (64.1)	137 (29.5)	24 (5.2)	6 (1.3)
	Request a prescription refill	238 (51.2)	183 (39.4)	35 (7.5)	9 (1.9)
	Request a referral to health care provider	120 (25.8)	260 (55.9)	62 (13.3)	23 (4.9)
**Billing and insurance**					
	View bill	327 (70.3)	88 (18.9)	35 (7.5)	15 (3.2)
	Pay bill	299 (64.3)	97 (20.9)	56 (12.0)	13 (2.8)
**Sharing and updating information**					
	Share record outside of health care system	154 (33.1)	248 (53.3)	48 (10.3)	15 (3.2)
	Download vaccination data as QR code	173 (37.2)	221 (47.5)	52 (11.2)	19 (4.1)
	Download full record, summary, or visit information	113 (24.3)	261 (56.1)	77 (16.6)	14 (3.0)
	Enter vaccine information	106 (22.8)	274 (58.9)	57 (12.3)	28 (6.0)
	Pull data from state vaccine registry	100 (21.5)	283 (60.9)	54 (11.6)	28 (6.0)
	Link information from record to a third-party app	61 (13.1)	227 (48.8)	147 (31.6)	30 (6.5)
	Match profile to potential research studies	38 (8.2)	252 (54.2)	141 (30.3)	34 (7.3)

**Table 2:** Desired features and data needs related to patient portals.

Desired features and data needs	Participants (n=465), n (%)
Share genetic testing information with health care providers	343 (73.8)
Access to test results before health care provider reviews them	341 (73.3)
Share living and social situation with health care providers	295 (63.4)
Assistance in portal use due to physical, sensory, cognitive disabilities	220 (47.3)
Assistance in portal use because of language barriers	200 (43.0)
Want data to be explained more clearly	157 (33.8)
Want more data from health care providers	135 (29.0)
Want better ways to view my data (eg, better graphs)	129 (27.7)
Want to upload own data from apps or wearable devices	90 (19.4)

## Discussion

The patient-centered care movement has expanded efforts to increase consumer engagement with EHI via patient portals and other digital health tools. In 2023, the most utilized portal features were viewing laboratory results and accessing visit information, indicating the features’ importance to users. However, features such as pulling vaccination data from state registries and medical record downloads, despite being of interest, were underutilized. About half of the respondents noted that they were interested in accessing EHI via third-party apps and matching their profile to potential research studies but had not used these features. Further, certain features such as having immediate access to test results and accessing EHI via third-party apps should be available to all patients due to the Cures Act Final Rule [5], suggesting a potential lack of awareness of certain functionalities. Together, these findings indicate opportunities to increase outreach to patients and educate both patients and providers on available features. Finally, this research provides insights into future data and accessibility needs.

This study demonstrates the importance of specific portal features and ongoing data needs. While these findings may not be generalizable to the US population, they provide important insights into the current state of portal use and the needs of a state’s population. Future research should replicate this study at the national level and in medically underserved communities to better understand diverse needs, as well as identify desired features that are not currently available in patient portals.

In conclusion, in 2023, based on a sample from one state’s population, most participants used patient portals, but the use of the 22 functions varied widely. This helps understand the demand for portal features and informs portal design. The increasing use of patient portals underscores the importance of elucidating data needs across a broad range of users, implementing desired features to increase patient engagement and ultimately achieve better health outcomes [9,10].

## Appendix

Multimedia Appendix 1 Study setting.

Multimedia Appendix 2 Survey questionnaire.

Multimedia Appendix 3 Methodology.

## Abbreviations

EHI: electronic health information
