# Supplementary material for: Understanding Patient Portal Uses and Needs: Cross-Sectional Study in a State Fair Setting
Source: JMIR Form Res. 2024 Oct 11;8:e64085. doi: 10.2196/64085 (PMC11512118; doi:10.2196/64085)

Appendix 1

Study Setting: Driven to Discover (D2D) Research Facility at the Minnesota State Fair

The Driven to Discover Research Facility (D2D) was launched by the University of Minnesota in 2014 to bring University research to the Minnesota State Fair.
“The mission of D2D is to:

- Promote greater citizen participation in research
- Showcase University of Minnesota research.
- Support fast, efficient subject recruitment.
- Provide access to a diverse participant pool.
- Provide the opportunity for unique studies and longitudinal tracking.
- Promote faculty and student engagement.
- Provide opportunities for cross-University collaboration in subject recruitment.”

Researchers benefit from D2D as it provides a venue to reach a more diverse pool of participants and meet recruitment goals in a few days.

Fairgoers benefit from D2D as it provides an option to participate in research in a convenient way along with the innovative approach of state fair setting.

Attendance in the 2023 Minnesota State Fair was 1,835,826 (compared to estimated state population of ~5.7 million in 2023). Over 55,000 fairgoers visited D2D building for research during the 2023 Fair and participated in 47 different studies. This study on patient portals was conducted in the 2023 Minnesota State Fair with 523 participants.

For more information: http://d2d.umn.edu/

Photos from 2023 Minnesota State Fair Study

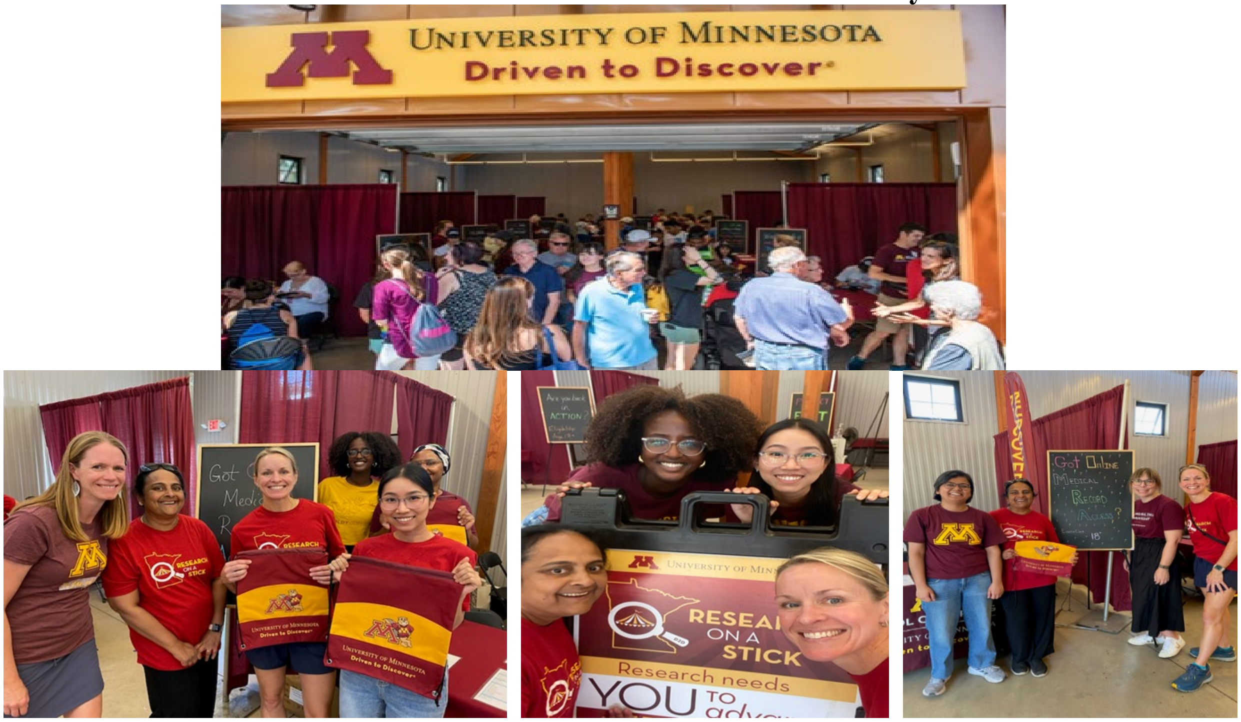

Supplement: Multimedia Appendix 1 [file formative_v8i1e64085_app1.pdf]
