# Supplementary material for: Understanding Patient Portal Uses and Needs: Cross-Sectional Study in a State Fair Setting
Source: JMIR Form Res. 2024 Oct 11;8:e64085. doi: 10.2196/64085 (PMC11512118; doi:10.2196/64085)
Supplement: Multimedia Appendix 2 [file formative_v8i1e64085_app2.pdf]

UNIVERSITY OF MINNESOTA
Driven to Discover®

Intro questions

Do you access your medical records online? Share your experience!

Consent:
Welcome to this research study.

A patient portal is a secure online website or mobile application that gives patients convenient, 24-hour access to personal health information from anywhere with an Internet connection. A healthcare provider or an organization that pays for healthcare services might have offered you access to a patient portal. You always have to login to the patient portal. Some examples of a patient portal are MyChart®, Healow by eClinicalWorks®, InteliChart®, or a login page branded with your healthcare provider's logo or name. You do not need to use a patient portal to participate in this study.

In this study, we will ask you questions about whether you access your health information online using a patient portal, what types of information you like to view in the patient portal, and what types of information you would like added to your patient portal. We will also ask you questions about how easy or hard patient portals are to use, to help improve their design. The study should take you around 10 minutes to complete. You will receive a drawstring backpack for your participation.

Your participation in this research is voluntary. You have the right to withdraw at any point during the study. Your answers are kept safe. Your unidentified data

will be used for research studies. The Principal Investigator of this study can be contacted at Sripriya Rajamani (sripriya@umn.edu).

The first 3 questions ask about your overall health.

In general, would you say your health is...

- Excellent
- Very good
- Good
- Fair
- Poor

Overall, how confident are you about your ability to take good care of your health?

- Completely confident
- Very confident
- Somewhat confident
- A little confident
- Not confident at all

Do you have any chronic health conditions (e.g., high blood sugar / high blood pressure / joint pains / breathing issues / depression) which require care by health professionals?

- Yes
- No

The following questions ask about online access to your medical records through patient portals.

Which clinics or health care systems do you generally go to for your healthcare needs (check all that apply)?

- ☐ Allina Health
- ☐ Altru Clinic
- ☐ Avera Medical Group
- ☐ Carris Health
- ☐ CentraCare
- ☐ Community-University Health Care Center
- ☐ Entira Family Clinics
- ☐ Essentia Health
- ☐ Hennepin Health/HCMC
- ☐ Health Partners/Park Nicollet
- ☐ Lakewood Clinics
- ☐ Mayo Clinic Health System
- ☐ MHealth Fairview Clinics
- ☐ North Memorial Clinics
- ☐ Olmsted Medical Center / Clinics
- ☐ Ridgeview clinics
- ☐ Sanford Health
- ☐ Stillwater Medical Group
- ☐ Tri-county Health Care
- ☐ University of Minnesota Physicians

- [ ] Other – please write the name of clinic / health system

---

Has a clinic, hospital, or health system ever offered you access to a patient portal?

- ○ Yes
- ○ No
- ○ Don't know

If yes, which clinic, hospital or health system has offered you access to a patient portal? (Check all that apply)

- [ ] Allina Health
- [ ] Altru Clinic
- [ ] Astera Health
- [ ] Avera Medical Group
- [ ] Carris Health
- [ ] CentraCare
- [ ] Community-University Health Care Center
- [ ] Entira Family Clinics
- [ ] Essentia Health
- [ ] Hennepin Healthcare (includes Hennepin County Medical Center (HCMC))
- [ ] Health Partners/Park Nicollet
- [ ] Lakewood Clinics

- ☐ Mayo Clinic Health System
- ☐ MHealth Fairview Clinics
- ☐ North Memorial Clinics
- ☐ Olmsted Medical Center / Clinics
- ☐ Ridgeview clinics
- ☐ Sanford Health
- ☐ Stillwater Medical Group
- ☐ Tri-county Health Care
- ☐ University of Minnesota Physicians
- ☐ Other – please write the name of clinic / health system

Have any of your doctors, nurses, or other healthcare workers or office staff members ever encouraged you to use a patient portal?

- ○ Yes
  If yes- who? Nurse/doctor/other healthcare worker/office staff/other professional?
- ○ No
- ○ Don't know

How many times did you login to your patient portal in the last 12 months?

- ○ 0
- ○ 1 to 2 times
- ○ 3 to 5 times
- ○ 6 to 9 times
- ○ 10 or more times

Why have you not accessed your medical record online? (Check all that apply)

Mark all that apply

- ☐ You don’t have access to a patient portal
- ☐ You prefer to speak to your health care provider directly
- ☐ You found it difficult to login or are not comfortable with computers
- ☐ You did not have a need to use your online medical record
- ☐ You were concerned about the privacy or security of online access

Demograhics

The last set of questions ask about you so that we can understand your personal experience.

What is your age?

- ○ 18-24
- ○ 25-44
- ○ 45-64
- ○ 65-84

- 85 and older

What is your current gender identity?

- Male
- Female
- Non-binary / third gender
- Transgender
- A gender not listed here
- Prefer not to say

What is your marital status?

- Married
- Living as married or living with a romantic partner
- Divorced
- Widowed
- Separated
- Single, never been married
- Prefer not to say

What would you consider to be your racial background?
<u>One or more categories may be selected. Mark all that apply.</u>

- [ ] White
- [ ] Black/African American
- [ ] Somali
- [ ] Asian
- [ ] Chinese
- [ ] Hmong

- [ ] Indian
- [ ] Lao
- [ ] Vietnamese
- [ ] Native American/Alaskan
- [ ] Native Hawaiian/Pacific islander
- [ ] Two or More Races
- [ ] Other
- [ ] Prefer not to say

Are you of Hispanic, Latino/a, or Spanish ethnicity?

- ○ Non-Hispanic/Latinx
- ○ Hispanic/Latinx
- ○ Prefer not to say

What is the highest grade or level of school you have completed?

- ○ Less than 8 years
- ○ 8 through 11 years
- ○ 12 years or completed high school
- ○ Post high school training other than college (vocational or technical)
- ○ Some college
- ○ College graduate
- ○ Postgraduate

Which one of these comes closest to your own feelings about your household's income?

- ○ Living comfortably on present income
- ○ Getting by on present income

- ○ Finding it difficult on present income
- ○ Finding it very difficult on present income

Would you describe where you live as

- ○ Suburban
- ○ Urban
- ○ Rural

End of survey questions

Thank you for your time and input.

Are you interested in participating in a future study related to this topic of portal use and preferences? This study will provide financial incentive for your participation.
If YES, we request you to share your name and email so that we can reach out. Please note that your email will NOT be linked to your survey responses provided in this study. This is completely voluntary; it is ok to end your participation now.

- ○ Yes
- ○ No

Thank you for your interest in participating in a future study related to this topic of portal use and preferences.

Name __________

Email __________

Please collect your UMN backpack and enjoy your time at the Fair 😊

If accessed patient portal questions

How did you access your online medical record?

- ○ App (on your phone or tablet)
- ○ Website (on your computer or phone)
- ○ Both app and website
- ○ Don't know

Finding care using patient portals

Please indicate your use of these features and your interest in using it. Please mark one response per row.

	Have used	Have NOT used it, but interested in using	Have NOT used it, and NOT interested in using	This feature is not available to me
Schedule urgent visit for a health condition	○	○	○	○
Schedule non-urgent appointment	○	○	○	○
Schedule e-visit or telehealth or video visit	○	○	○	○

Communication using patient portals

Please indicate your use of these features and your interest in using it. Please mark one response per row.

	Have used	Have NOT used it, but interested in using	Have NOT used it, and NOT interested in using	This feature is not available to me
Send a message to your doctor, nurse, or other member of your care team to ask a question	○	○	○	○
Send a message to your doctor, nurse, or other member of your care team to provide them information	○	○	○	○
Request a prescription refill	○	○	○	○
Request a referral to another healthcare provider	○	○	○	○

View information from your patient portal

Please indicate your use of these features and your interest in using it. Please mark one response per row.

	Have used	Have NOT used it, but interested in using	Have NOT used it, and NOT interested in using	This feature is not available to me
View your medications	○	○	○	○
View your vaccinations	○	○	○	○
View your lab results	○	○	○	○
View prior and upcoming visit	○	○	○	○

	Have used	Have NOT used it, but interested in using	Have NOT used it, and NOT interested in using	This feature is not available to me
information				
Complete questionnaires and forms	○	○	○	○
Complete advance care planning documents	○	○	○	○

Billing and Insurance

Please indicate your use of these features and your interest in using it. Please mark one response per row.

	Have used	Have NOT used it, but interested in using	Have NOT used it, and NOT interested in using	This feature is not available to me
View your bill	○	○	○	○
Pay your bill online	○	○	○	○

Sharing and Updating Information using Patient Portals

Please indicate your use of each feature and your interest in using it. Please mark one response per row.

	Have used	Have NOT used it, but interested in using	Have NOT used it, and NOT interested in using	This feature is not available to me
**Sharing your medical record with health care providers outside of your**	○	○	○	○

	Have used	Have NOT used it, but interested in using	Have NOT used it, and NOT interested in using	This feature is not available to me
**health care system.** This could be helpful when seeing specialists at other organizations, during emergency treatment, or while traveling.				
**Linking information from your medical record with a 3rd party app** so you can do extra things with your health care information. For example, you might link your clinic blood pressure values to a medication tracking app.	○	○	○	○
**Matching your profile to potential research studies** to receive information on research studies and to be contacted for research studies that you may be able to enroll in.	○	○	○	○
**Downloading your full medical record, health summary, or visit-specific information**. This could include a human-readable file and a file that other computer systems could read.	○	○	○	○
**Downloading your vaccination data,**	○	○	○	○

	Have used	Have NOT used it, but interested in using	Have NOT used it, and NOT interested in using	This feature is not available to me
**such as COVID vaccines, as a QR code.** Any health care organization or other authorized organization who needs to know your vaccine history could scan the QR code to get it.				
**Pulling information from the state immunization/vaccine registry into your portal.** This could be helpful if you received any vaccine, including a COVID vaccine, outside of your healthcare system	○	○	○	○
**Entering your own vaccine information, such as COVID vaccines, into your portal.** You could enter missing vaccine information, or correct errors.	○	○	○	○

Would you like any of the following features in a patient portal?

	Yes	No
Access to test results before your healthcare provider reviews them	○	○

	Yes	No
Ability to share genetic testing information with healthcare providers	○	○
Ability to share living and social situation (worrying about food, forced to move, transportation issues etc.) with healthcare providers	○	○
Assistance in using the patient portal because of language barriers	○	○
Assistance in using the patient portal because of physical, sensory, and cognitive disabilities	○	○

Are you currently caring for or making health care decisions for someone with a medical, behavioral, disability, or other condition?

- ○ Yes, a child/children
- ○ Yes, a spouse/partner
- ○ Yes, a parent/parents
- ○ Yes, another family member
- ○ Yes, a friend or other non-relative
- ○ No

Do you have access rights (i.e., permission to log-in) to the patient portal as a parent/caregiver/family/friend?

- ○ No

- Yes – approved access from their healthcare provider
- Yes – shared user ID and password

Do you have anyone who provides care for you and helps you in making health care decisions?

- Yes, a child/children
- Yes, a spouse/partner
- Yes, a parent/parents
- Yes, another family member
- Yes, a friend or other non-relative
- No

Have you given access rights (i.e., permission to log-in) to your patient portal to that caregiver or a family/friend?

- No
- Yes – approved access from your healthcare provider
- Yes – shared user ID and password

Are there any features or information you do NOT currently have access to in your patient portal that you would like to have access to?

- ○ Yes
  If yes, please note below. This can include your role as a parent or a caregiver.
- ○ No

Do you find it easy to use your patient portal?

Very easy	Somewhat easy	Somewhat difficult	Very difficult
○	○	○	○

Do you find it easy to understand the information in your patient portal (e.g., test results, medications, etc.)?

Very easy	Somewhat easy	Somewhat difficult	Very difficult
○	○	○	○

What suggestions do you have for improving your patient portal? (Check all that apply)

- ☐ I want more data from my health care providers
- ☐ I want to upload my own data from apps or wearable devices
- ☐ I want better ways to view my data (for example, better graphs)
- ☐ I want my data to be explained more clearly

☐ Please write any other suggestion(s)

Powered by Qualtrics
