# Supplementary material for: Understanding Patient Portal Uses and Needs: Cross-Sectional Study in a State Fair Setting
Source: JMIR Form Res. 2024 Oct 11;8:e64085. doi: 10.2196/64085 (PMC11512118; doi:10.2196/64085)
Supplement: Multimedia Appendix 3 [file formative_v8i1e64085_app3.pdf]

Appendix 3
Study Methodology

- Study data collected (n=523) from the D2D facility in 2023 Minnesota State Fair was analyzed using the statistical software SPSSv28.
- Preliminary analysis conducted on overall participants (n=523) and their demographic data.
- Subsequently, the analysis focused on just the portal users (n=465, 89%).
- Demographic characteristics of the portal users was analyzed (race, ethnicity, gender, marital status, education, urban/rural residence, income).
- This was followed by analysis of 22 features along with 4 options to indicate interest and use
    - (a) Have used; (b) Have NOT used, but interested in using; (c) Have NOT used, and NOT interested in using; (d) Feature not available

1. View lab results
2. View prior and upcoming visit info
3. Complete questionnaires and forms
4. View vaccinations
5. View medications
6. Complete advanced care planning
7. Schedule non-urgent appointment
8. Schedule e-visit or telehealth or video visit
9. Schedule urgent visit for a health condition
10. Ask question to doctor/nurse/care team
11. Provide info to doctor/nurse/care team
12. Request a prescription refill
13. Request a referral to healthcare provider
14. View bill
15. Pay bill
16. Share record outside of healthcare system
17. Download vaccination data as QR code
18. Download full record, summary or visit info
19. Enter vaccine information
20. Pull data from state vaccine registry
21. Link info from record to a 3rd party app
22. Match Profile to potential research studies

- These results were synthesized and are presented in Table 1
- Next set of analysis focused on desired features and data needs related to patient portals
    - ✓ Share genetic testing information with healthcare providers
    - ✓ Access to test results before healthcare provider reviews them
    - ✓ Share living and social situation with healthcare providers
    - ✓ Assistance in portal use due to physical, sensory, cognitive disabilities
    - ✓ Assistance in portal use because of language barriers
    - ✓ Want data to be explained more clearly
    - ✓ Want more data from health care providers
    - ✓ Want better ways to view my data (e.g., better graphs)
    - ✓ Want to upload own data from apps or wearable devices
- These results are formatted and presented in Table 2.
